# Catarrh Snuff

**Published:** 1874-12

**Authors:** 


CATARRH SNUFF.—Dr. E. C. Mann, of New York, recommends the use of a snuff composed of equal parts of finely pulverized camphor and white powdered sugar, as an adjuvant to the various injections and sprays employed in the treatment of nasal catarrh.—*New York Medical Journal.*

HE that will seek the truth earnestly, with patience and humility, in the fear of God and love of man, will surely find it. *Sir James Paget.*

